# Gli1 regulates stemness characteristics in gastric adenocarcinoma

**DOI:** 10.1186/s13000-020-00949-5

**Published:** 2020-05-19

**Authors:** Wenbo Qi, Zhaoting Yang, Ying Feng, Haoyue Li, Nan Che, Lan Liu, Yanhua Xuan

**Affiliations:** 1 Institute for Regenerative Medicine, Yanbian University College of Medicine, Yanji, China; 2 Department of Surgical Oncology, Lanzhou University Second Hospital, Lanzhou, 730000 China; 3 Department of Pathology, Yanbian University College of Medicine, Yanji, China; 4 Department of Pathology, Affiliated Hospital of Yanbian University, Yanji, China

**Keywords:** Glioma-associated oncogene homolog 1, Gastric adenocarcinoma, Cancer stemness, Prognosis

## Abstract

**Background:**

Glioma-associated oncogene homolog 1 (Gli1), affects the progression and the stemness characteristics of malignant carcinoma. The aim of the present study was to identify the relation between Glioma-associated oncogene homolog 1 (Gli1) and stemness and determine its clinical significance in gastric adenocarcinoma (GA). We investigated Gli1 expression and its correlation with other stemness-associated proteins in 169 GA samples and 5 GA cell lines.

**Methods:**

To elucidate the role of Gli1 in the clinicopathological significance and stemness of GA, tissues samples from 169 GA patients were collected for immunohistochemistry (IHC). Additionally, MKN74, MKN28, NCI-N87, SNU638, AGS cells were collected for western blotting, MKN28 cells were collected for spheroid formation assay.

**Results:**

Results showed that Gli1 expression was closely related to tumor grade, primary tumor (pT) stage, distant metastasis, clinical stage, gross type, microvessel density, and shorter overall survival (OS). Cox regression analysis verified that Gli1 was an independent prognostic factor for OS. Furthermore, Gli1 expression correlated with the expression of stemness-related genes, CD44, LSD1, and Sox9. Gli1 inhibitor GANT61 significantly decreased the expression of CD44 and LSD1, and spheroid formation ability of the MKN28 cells.

**Conclusions:**

In conclusion, Gli1 may be a poor prognostic indicator and a potential cancer stemness-related protein in GA.

## Background

As one of the common digestive tract tumors, gastric adenocarcinoma (GA) poses a serious threat to the health of patients across the world, especially in Asian countries. It was found that the 5-year survival rate for patients with early stage GA is approximately 90%, but it decreases to 16.6% for patients with advanced GA [1]. Although there are many treatments for advanced GA, the overall survival (OS) is still poor. Therefore, it is necessary to improve the current therapeutic modalities and to explore new biomarkers for predicting the progression of GA, thereby advancing in targeted therapies.

Cancer stem-like cells (CSCs) maintain the viability of the cancer cell population through self-renewal and infinite proliferation, and play an important role in survival, proliferation, metastasis, and tumor recurrence. CSCs are viewed as novel therapeutic targets due to their stemness potential [2]. Thus, understanding the molecular mechanisms of CSCs in GA initiation and progression may help elucidate the pathogenesis of GA.

The Hedgehog (Hh) signaling pathway comprises the Hh ligand (SHh, IHh, and DHh); twelve-transmembrane protein receptor, Patched (Ptc); seven- transmembrane protein receptor, Smoothened (Smo); Glioma-associated oncogene (Gli) family of transcription factors; and downstream target genes. The PTC gene includes the two homologs, PTCH1 and PTCH2, and both gene products can bind to Hh ligands. In the absence of the Hh ligand, PTCH inhibits Smo activity. The presence of Hh ligand relieves this inhibition, allowing smo to activate the Gli family of transcription factors. Gli1 has been implicated in several human cancers, including a role in the progression of pancreatic cancer [3] and an association with poor prognosis in glioblastoma [4], pancreatic cancer [5], and breast cancer [6]. Gli1 expression correlates with stemness in breast and lung cancers and is essential in the cellular proliferation and growth of these tumors [7, 8]. Although Gli1 expression has been studied in many human cancers, its role as a prognostic indicator and its functional significance in determining the stemness of GA cells warrants further investigation.

In this study, we investigated the clinicopathological value of Gli1 and evaluated the correlation between Gli1 and stemness in GA.

## Materials and methods

### Tissues

One hundred sixty-nine cases GA tissue in paraffin section were gained from the Affiliated Hospital of Yanbian University and comply with agreements approved by the institutional review committee. The tissue samples were collected from 1995 to 2000. Preoperative chemotherapy or radiotherapy was not implemented. Clinical and pathological reports were reviewed for age, sex, tumor size, tumor grade, tumor location, primary tumor (pT) stage, lymph node metastasis, distant metastasis, gross type and histological type.

### Cell lines

MKN74, MKN28, NCI-N87, SNU638, and AGS, were bought from the ATCC and were maintained in 1640 contained with 10% fetal bovine serum (FBS, Life Technologies, Grand Island, NY), 100 mg/ml penicillin G and 50 mg/ml streptomycin (Life Technologies, Grand Island, NY) at 37 °C in a humidified atmosphere containing 5% CO_2_. MKN28 and MKN74 cells were treated with corresponding GANT61 (GAN, ENZO Lifesciences).

### Immunohistochemical (IHC) staining procedure

After routinely dewaxing and hydration, sections proceed to be antigen repaired with TE buffer at 98 °C. Each section was blocked with 3% H_2_O_2_. Each section was incubated with anti-Gli1 (Abcam), anti-CD44 (Abcam), anti-LSD1 (Sigma), anti-Sox2 (R&D), anti-Sox9 (Abnova), anti-LGR5 (Abcam), in primary antibody dilution buffer for 1 h at ambient temperature (AT). Then anti-mouse/rabbit antibody were used to incubated with tissue samples for 30 min at AT. Lastly, chromogenic agent 3, 3′-diaminobenzidine (Dako) was used to stain tissue samples.

The double immunostaining procedure was executed in the same section, the first step was to use anti-Gli1 antibody staining with 3,3′-diaminobenzidine, the second step was to use anti-CD105 antibody (Abcam) staining with AEC.

Two pathologists (WB Qi & YH Xuan) assessed the immunohistochemical results and the staining results were assessed according to previous study [9].

### Western blotting

The tumor cells were lysed by using RIPA buffer with Phenyl methane sulfonyl fluoride (PMSF). The same amount of protein was separated with 10% SDS-PAGE gel and then was transferred onto PVDF membranes (Biotech). Subsequently, 5% skim milk (diluted in PBS) was used to blocked the PVDF membranes for 2 h at RT. And then the membranes were incubated with anti-Gli1 (Santa), anti-CD44 (Abcam), anti-LSD1 (ZSGB-BIO), anti-Sox9 (Abcam), anti-β-actin (Abcam). The next step is to incubate anti-rabbit/mouse for 2 h. Detection was performed by the ECL kit.

### Immunofluorescence analysis

MKN28 cells were planted and were cultured to 60–70% density. 4% polyformaldehyde was used to fix cells for 20 min. And 0.5% Triton X-100 was used to permeabilize cells for 20 min. Next, 3% BSA was used to block cells for 1 h. Absorbent paper absorbs the sealing liquid and does not wash. Cells were incubated with anti-Gli1/LSD1, anti-Gli1/Sox9 for 2 h. The next day, cells were incubated with second-fluorescence antibodies (Invitrogen, A12380 and A11008) for 1 h. Finally, DAPI was used to stain the nuclear. Fluorescence detection was performed with the Axiovert200II (Carl-Zeiss).

### Tumorsphere-forming assay

MKN28 cells were maintained in serum-free DMEM medium (Invitrogen) with EGF (Pepro Tech), bFGF (Pepro Tech), B27 (GIBCO), heparin (Sigma), penicillin and streptomycin. Subsequently, cells were planted in low attachment culture dishes (Corning). After 1 week and 2 weeks, light microscopy was used to examine cell morphology.

### Statistical analysis

SPSS 25.0 statistical software (NO. 1975–01566-C), Pearson’s chi-square test and mean ± standard deviation was used for the data analysis, and the results was evaluated by analysis of variance. The Kaplan-Meier method was used to identify the overall survival (OS) and were compared using the log-rank test. Univariate and multivariate analysis was used for the Cox proportional hazards model. The GraphPad Prism 7 software is used for statistics on the results of western blotting. *P* value less than 0.05 was considered to have statistical significance.

## Results

### Association between the expression of Gli1 and clinical characteristics of GA

**Fig. 1:**
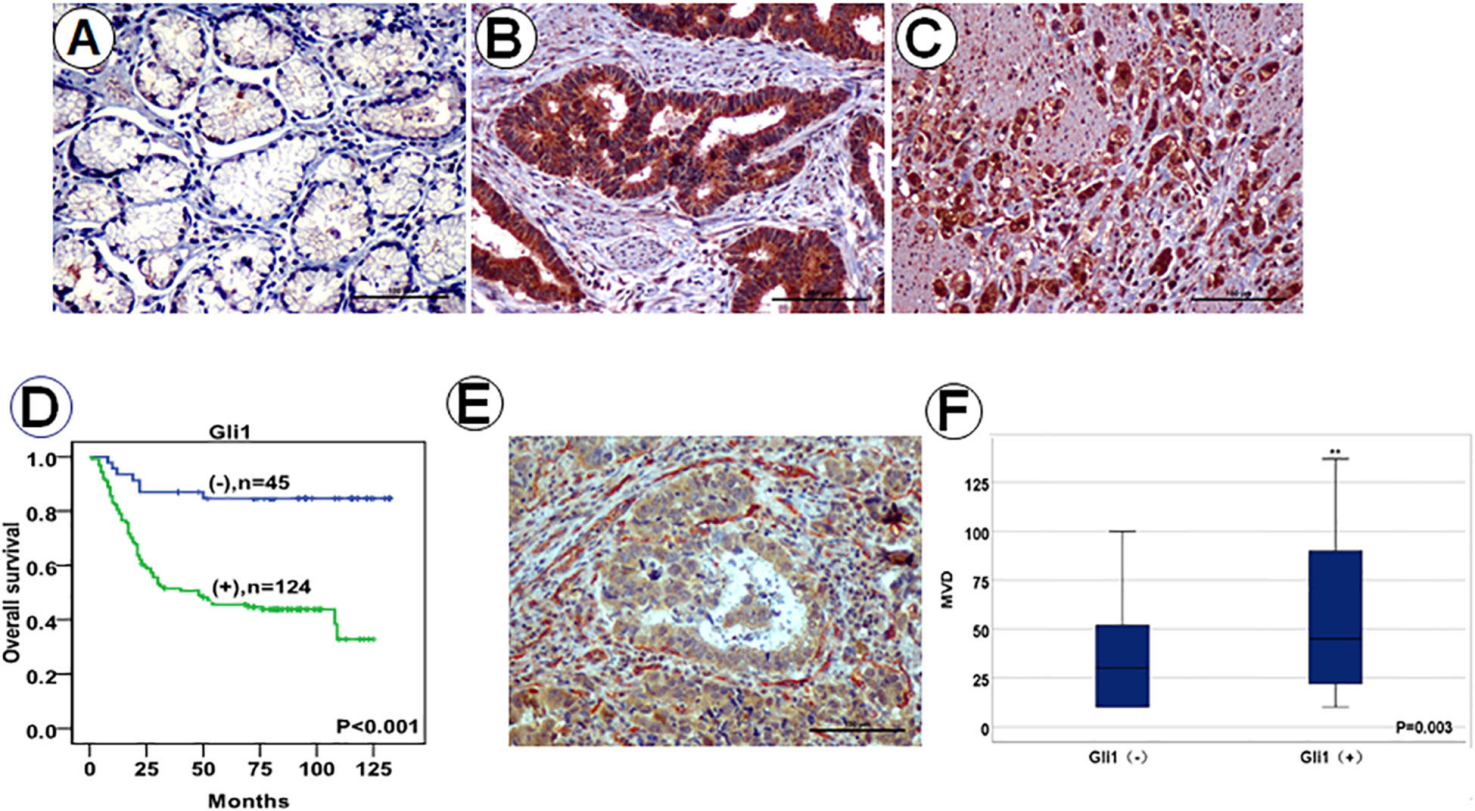
Gli1 is associated with unfavorable clinicopathological parameters in GA. Immunohistochemical staining of Gli1 expression in normal gastric epithelium tissues (**a**), moderate differentiated GA (**b**), poor differentiated GA (**c**). The positive expression of Gli1 in GA was significantly associated with a shortened OS compared to the negative groups (**d**). Images of immunohistochemical double staining for Gli1/CD105 in GA tissues (Gli1: brown reaction product; CD105: red reaction product) (**e**). Expression of Gli1 in GA was significantly associated with increased microvessel density (MVD) (**f**)

To understand if Gli1 is associated with GA progression, we investigated Gli1 expression in human GA by a Tissue Microarray (TMA) analysis. TMA analysis was performed for Gli1 expression by IHC staining in adjacent non-tumorous gastric epithelium and GA tissues. IHC staining revealed that Gli1 expression in GA (Fig. 1b-c) was higher than non-tumorous gastric epithelium (Fig. 1a). Gli1 significantly correlated with tumor grade (*P* = 0.001), pT stage (*P* = 0.029), clinical stage (*P* = 0.005), distant metastasis (*P* = 0.007), and gross type (*P* = 0.021) (Table 1), not with age, sex, tumor location, tumor size, lymph node metastasis, histological type. Interestingly, our results find a correlation between Gli1 expression and pT stage and distant metastasis, but no correlation with tumor size or lymph node metastasis. These results are accordance with the data in GEPIA (Gene Expression Profiling Interactive Analysis) and TCGA (The Cancer Genome Atlas) that Gli1 expression was higher in clinical stage (2/3/4) compared with clinical stage (1) (*P* < 0.001), and was not correlated with lymph node metastasis (Supplemental Figure).

**Table 1:** Comparison of clinicopathologic characteristics according to the Gli1 expression in GA

Variable	N	Gli1(−)n (%)	Gli1(+)n (%)	χ2	*R*	*P*
Age (years)				1.448	0.093	0.229
< 65	109	32(29.4)	77(70.6)			
≥ 65	60	13(21.7)	47(78.3)			
Sex				1.347	0.089	0.246
Male	106	25(23.6)	81(76.4)			
Female	63	20(31.7)	43(68.3)			
Tumor size (cm)				0.759	0.065	0.384
< 4.5	63	19(30.2)	44(69.8)			
≥ 4.5	106	26(24.5)	80(75.5)			
Tumor grade				15.771	0.049	0.001*
Well	31	14(45.2)	17(54.8)			
Moderate	66	11(16.7)	55(83.3)			
Poor	72	20(27.8)	52(72.2)			
Tumor location				1.943	0.024	0.584
Antrum	93	23(24.7)	70(75.2)			
Cardia	3	0(0)	3(100.0)			
Body	63	20(31.7)	43(68.3)			
Mix	10	2(20.0)	8(80.0)			
pT stage				9.034	0.218	0.029*
1	35	16(45.7)	19(54.3)			
2	38	11(28.9)	27(71.1)			
3	92	17(18.5)	75(81.5)			
4	4	1(25.0)	3(75.0)			
Lymph node metastasis				1.949	0.105	0.163
Negative	144	41(28.5)	103(71.5)			
Positive	25	4(16.0)	21(84.0)			
Distant metastasis				7.403	0.208	0.007*
Negative	151	45(29.8)	106(70.2)			
Positive	18	0(0)	18(100.0)			
Clinical stage				12.799	0.262	0.005*
1	44	18(40.9)	26(59.1)			
2	34	11(32.4)	23(67.6)			
3	73	16(21.9)	57(78.1)			
4	18	0(0)	18(100.0)			
Gross type				5.365	0.177	0.021*
Early gastric cancer	37	16(43.2)	21(56.8)			
Advanced gastric cancer	132	29(22.0)	103(78.0)			
Histological type				0.389	0.032	0.823
Intestinal	91	26(28.6)	65(71.4)			
Diffuse	70	17(24.3)	53(75.7)			
Mix	8	2(25.0)	6(75.0)			
Survival				23.883	0.375	< 0.001*
Die	78	7(9.0)	71(91.0)			
Alive	91	38(41.8)	53(58.2)			

*Statistically significant findings

The Kaplan-Meier survival analysis revealed that Gli1 expression in GA was associated with lower OS (*P* < 0.001; Fig. 1d). The univariate Cox regression analysis showed that tumor size, pT stage, lymph node metastasis, distant metastasis, and Gli1 expression (all *P* < 0.05) were independent prognostic factors for poor OS. The multivariate Cox regression analysis revealed that pT stage, lymph node metastasis, distant metastasis, and Gli1 expression (all *P* < 0.05) were independent prognostic predictors for OS (Table 2). These results demonstrated that Gli1 is a potential prognostic biomarker of GA.

**Table 2:** Univariate and multivariate analyses of prognostic variables for overall survival in GA patients using Cox proportional hazards regression

Characteristic	Univariate analyses			Multivariate analyses		
	HR	95% CI	*P*	HR	95% CI	*P*
Age (years)			0.202			0.494
< 65	1.00		–	1.00		–
≥ 65	1.326	0.859–2.044		1.178	0.737–1.882	
Tumor size (cm)			0.007*			0.888
< 4.5	1.00			1.00		
≥ 4.5	1.918	1.190–3.091		1.042	0.591–1.835	
pT stage			< 0.001*			< 0.001*
1	1.00			1.00		
2	4.944	1.440–16.974		1.639	1.154–22.990	
3	12.358	3.875–39.412		2.352	2.472–44.689	
4	20.989	4.690–93.938		3.854	7.152–311.452	
Lymph node metastasis			< 0.001*			< 0.001*
Negative	1.00		–	1.00		–
Positive	5.610	3.446–9.133		3.272	1.908–5.613	
Distant metastasis			< 0.001*			0.002*
Negative	1.00		–	1.00		–
Positive	5.050	2.952–8.640		2.528	1.416–4.513	
Gli1			< 0.001*			0.002*
Negative	1.00		–	1.00		–
Positive	5.245	2.401–11.458		3.572	1.573–8.112	

*Statistically significant findings

Furthermore, double-staining results proved that CD105 expression (blood vessels) was around Gli1 expression (cancer cells) (Fig. 1e). Microvessel density (MVD) was significantly higher in Gli1(+) group (55.51 ± 36.34) than in Gli1(−) group (36.86 ± 30.85) (*P* = 0.003; Fig. 1f). These results demonstrated that Gli1 may be likely to metastasize through the microangiogenesis and then promoting distant metastasis and finally promote tumor progression. This result further explained the potential reasons why there is an association between Gli1 and distant metastasis.

### Correlation between Gli1 and stemness in GA

**Fig. 2:**
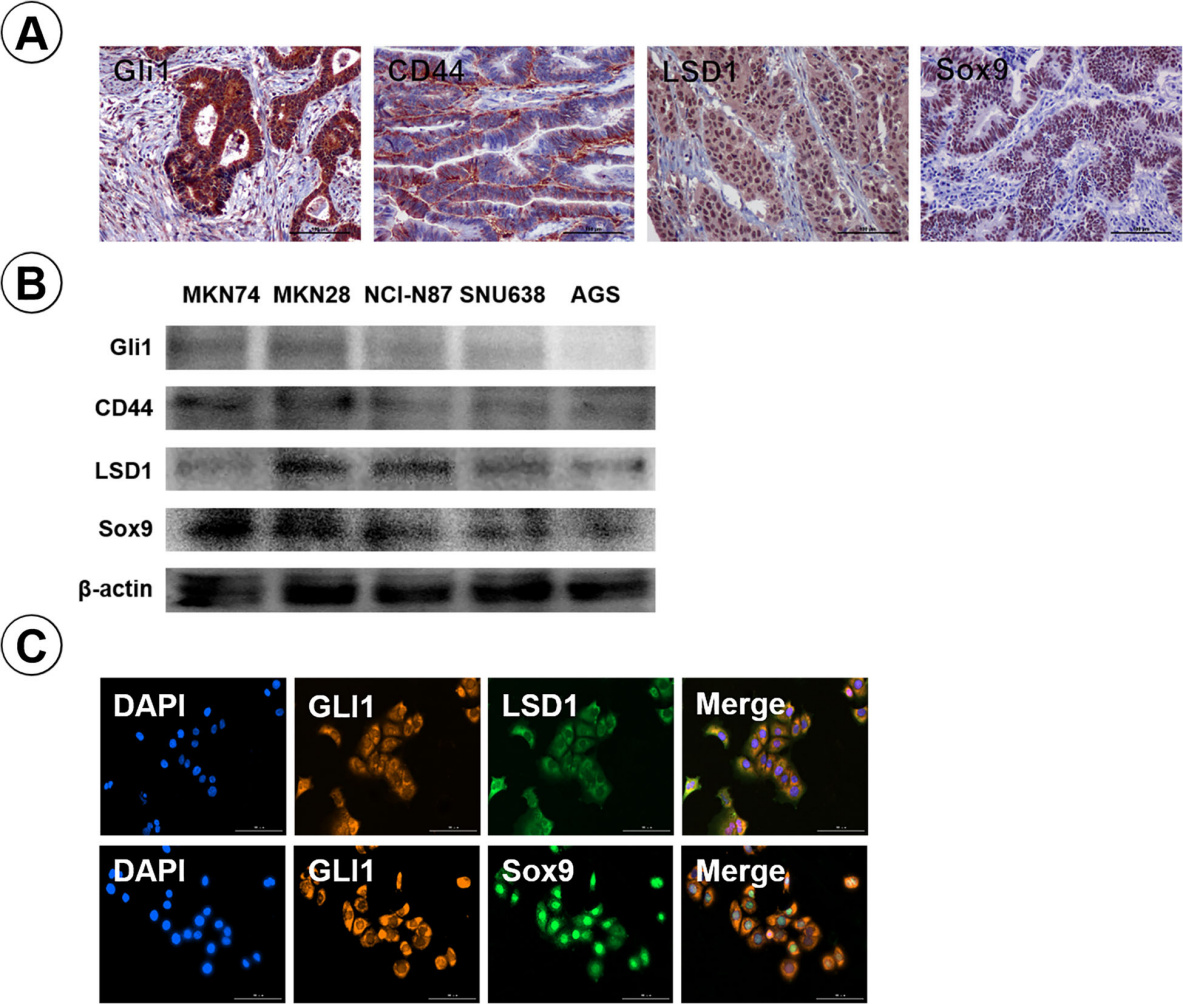
Gli1 is associated with stemness of GA. **a** Immunohistochemical staining of Gli1, CD44, LSD1, and Sox9 in the same field of GA tissues. (Original magnification×200). **b** Western blotting analysis of Gli1 and stemness related genes CD44, LSD1, and Sox9 in GA cell lines. **c** Immunofluorescence analysis of Gli1, LSD1, and Sox9 in MKN28 cell

We have reported that Gli1 is associated with stemness in breast cancer and lung squamous cell carcinoma [9, 10]. To identify the role of Gli1 in cancer stemness of GA, we studied Gli1 and stemness-related protein expression in GA. The result showed that Gli1 related with cancer stemness proteins, CD44, LSD1, and Sox9 (all *P* < 0.05) (Fig. 2a) (Table 3). Gli1, LSD1, Sox9 were primarily expressed in the nucleus of cancer cells; CD44 primarily located in the membranes of cancer cells. To further confirm above results, we investigated Gli1, CD44, LSD1, Sox9 expression in GA cells by western blotting. Gli1 expression in GA cells (MKN74, MKN28, NCI-N87, SNU638, AGS) were similar with stemness–related proteins (Fig. 2b). Furthermore, an immunofluorescence assay indicated that the Gli1-positive cell population were strongly identical with the LSD1 and Sox9 positive cell population within the MKN28 cells (Fig. 2c).

**Table 3:** Correlation of Gli1 expression with cancer stemness related proteins expression in GA

Variable	N	Gli1(−) n(%)	Gli1(+) n(%)	χ^2^	*R*	*P*
CD44				5.595	0.178	0.018*
Negative	2	2(100.0)	0(0)			
Positive	167	43(25.7)	124(74.3)			
LSD1				17.866	0.318	< 0.001*
Negative	58	27(46.6)	31(53.4)			
Positive	111	18(16.2)	93(83.8)			
Sox2				3.057	0.132	0.080
Negative	5	3(60.0)	2(40.0)			
Positive	164	42(25.6)	122(74.4)			
Sox9				12.748	0.271	< 0.001*
Negative	7	6(85.7)	1(14.3)			
Positive	162	39(24.1)	123(75.9)			
LGR5				1.869	0.105	0.172
Negative	83	26(31.3)	57(68.7)			
Positive	86	19(22.1)	67(77.9)			

*Statistically significant findings

**Fig. 3:**
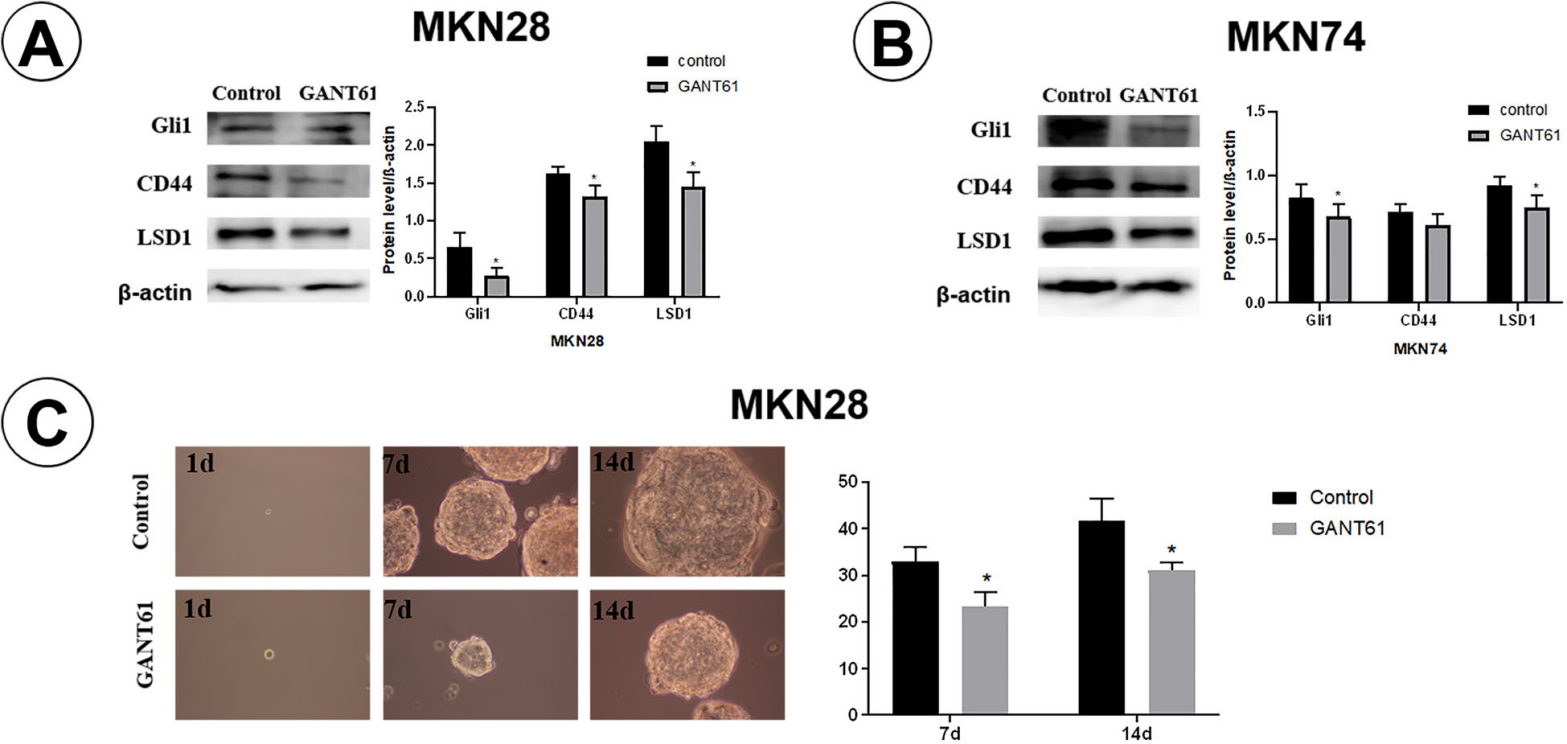
GANT61 decreases stemness of GA. **a**-**b** Western blotting analysis of Gli1, CD44, LSD1 in control group and GANT61 group, β-actin was used as a loading control. **c** Clonogenic proliferation of MKN28 cells in control group and GANT61 group (original magnification× 200). The number of tumorspheres/3000 cells was counted. (*: *P* < 0.05, *n* = 3)

To further understand the interaction between Gli1 and cancer stemness in GA cells, we blocked Gli1 expression using Gli1 inhibitor in MKN28 and MKN74 cells. Our studies showed that protein CD44, LSD1, Sox9 expression in MKN28 and MKN74 cells were significantly decreased after Gli1 was inhibited (*P* < 0.05, Fig. 3a-b). Subsequently, tumorsphere-forming experiment was performed to investigate the ability of clonogenic potential of MKN28 cells. Notably, MKN28 cells dealed with GANT61 reduced clonogenic potential compared with cells treated with DMSO (control group) (Fig. 3c). These results indicate a possibility that expression of Gli1 may enhances cancer cells to acquire stemness properties thereby promoting progression of GA.

## Discussion

Gli1 promotes the progression of many types of cancers including, pancreatic and prostate cancer [10–12]. Our study showed that Gli1 is overexpressed in GA tissue samples, and its expression correlates with adverse clinicopathological parameters. This suggests a role for Gli1 in the initiation, progression, and metastasis of GA. In addition, Gli1 expression correlates with unfavorable prognosis of breast cancers [13–15]. Our survival analysis revealed similar poor prognosis correlated with Gli1 overexpression in GA. Angiogenesis is an important step in malignant tumor growth and progression [16]. Abnormally activated Hh/Gli1 pathway in gliomas promotes tumor microvessel formation [17], and Gli1 overexpression in esophageal tumors significantly correlates with increased microvessel density [18]. Similarly, Gli1 expression in our study was also associated with higher microvessel density, indicating that Gli1 may promote the progression of GA via angiogenesis.

In human gliomas, the Hh/Gli1 pathway plays an important role in CSC self-renewal and tumorigenicity [19]. Gli1 expression also significantly correlates with stemness characteristics of esophageal carcinoma [18]. Moreover, some of stemness related proteins were used to identify the gastric CSC populations, such as CD44, CD133, LSD1, Sox9, LGR5 [20–25]. Evidence exists for the presence of cancer stem cells in colorectal cancer, with some phenotypes being CD44+/CD166+ enriched CSCs [26]. Our study demonstrated that Gli1 expression correlates with expression of cancer stemness-related proteins. Colocalization of Gli1 with cancer stemness-related proteins in GA tissue indicates that Gli1 may be an important stemness-related protein in GA. Furthermore, stemness-related protein expression in MKN28 and MKN74 cell lines and the ability of MKN28 cell lines to form spheroids were diminished after Gli1 inhibitor GANT61 was used. These factors prove that Gli1 regulates specific features of CSCs, such as self-renewal and proliferation. However, further investigation is necessary to elucidate the mechanism of Gli1 action in GA stem cells.

The SHh signaling pathway is a major regulator of tumorigenesis, tumor progression and therapeutic response. Downstream effectors of the SHh pathway include Smo and Gli family of zinc finger transcription factors. Both are regarded as important targets for cancer therapeutics. SMO inhibition prevents the downstream activation of Gli transcription factors, leading to suppression of those genes associated with cancer growth and progression. To date, SMO inhibitors include cyclopamine [27], LDE225 [28], and GDC-0449 [29] were investigated in GC. Gli1 is an extremely important part of the Hh signaling pathway and can activate most of the Hh pathway target genes. Developing Gli-targeted approach has its merit because of the fact that Gli proteins can be activated by both SHh ligand-dependent and -independent mechanisms. Gli1 and Gli2 inhibitor include GANT 61 [30] and Arsenic Trioxide [31] that have shown potent inhibition of Gli1 and Gli2 in many cancer cell lines, one of these is GC cells. Currently, many preclinical studies and clinical trials are being conducted to evaluate the efficacy of this exciting class of targeted therapy in a variety of cancers. We expect these inhibitors to be used clinically to help GC patients with targeted therapies.

Generally, targeting cancer cell stemness-associated genes may be an effective therapeutic strategy to overcome tumor relapse and chemoresistance. In this study, Gli1 proved to be a molecular marker for cancer stemness and a prognostic indicator of GA. We speculate that jointly targeting Gli1 and other cancer stemness biomarkers will provide a novel vision to treat GA.

## Conclusions

Gli1 was upregulated in GA tissues and cancer cells and correlated with poor prognosis in GA patients. Knocking down Gli1 by specific inhibitor suppressed the expression of Gli1 protein levels. Reduced expression of Gli1 downregulated the protein levels of cancer stemness biomarkers while also decreasing cell clonogenic potential in GA cells. Thus, Gli1 may promote the progression of GA by maintaining GA cell stemness potential. Hh/Gli1 pathway may play an important role in CSC self-renewal and tumorigenicity. Taken the above results together, we speculated that Gli1 may play a potential role in cancer stemness and thus to accelerate the progression in GA.

### Limitations of this current study

Our study has several innate limitations to note. First, Spheroid formation experiments were performed only on the MKN28 cell line. Immunofluorescence was performed only on Gli1, LSD1, Sox9. Other stemness related features have not been tested.

## Supplementary information

**Additional file 1: Supplemental Figure.** The association between Gli1 mRNA expression and clinical stage, lymph node metastasis in GEPIA and TCGA data in GA.

## Data Availability

All data generated or analyzed during this study are included in this published article.

## Abbreviations

GA: Gastric adenocarcinoma
OS: Overall survival
CSCs: Cancer stem-like cells
Hh: Hedgehog
Ptc: Patched
Smo: Smoothened
Gli: Glioma-associated oncogene
pT: primary tumor
IHC: Immunohistochemical
PMSF: Phenyl methane sulfonyl fluoride
TMA: Tissue Microarray
