# Supplementary figures and images for: Gli1 regulates stemness characteristics in gastric adenocarcinoma
Source: Diagn Pathol. 2020 May 19;15:60. doi: 10.1186/s13000-020-00949-5 (PMC7236965; doi:10.1186/s13000-020-00949-5)

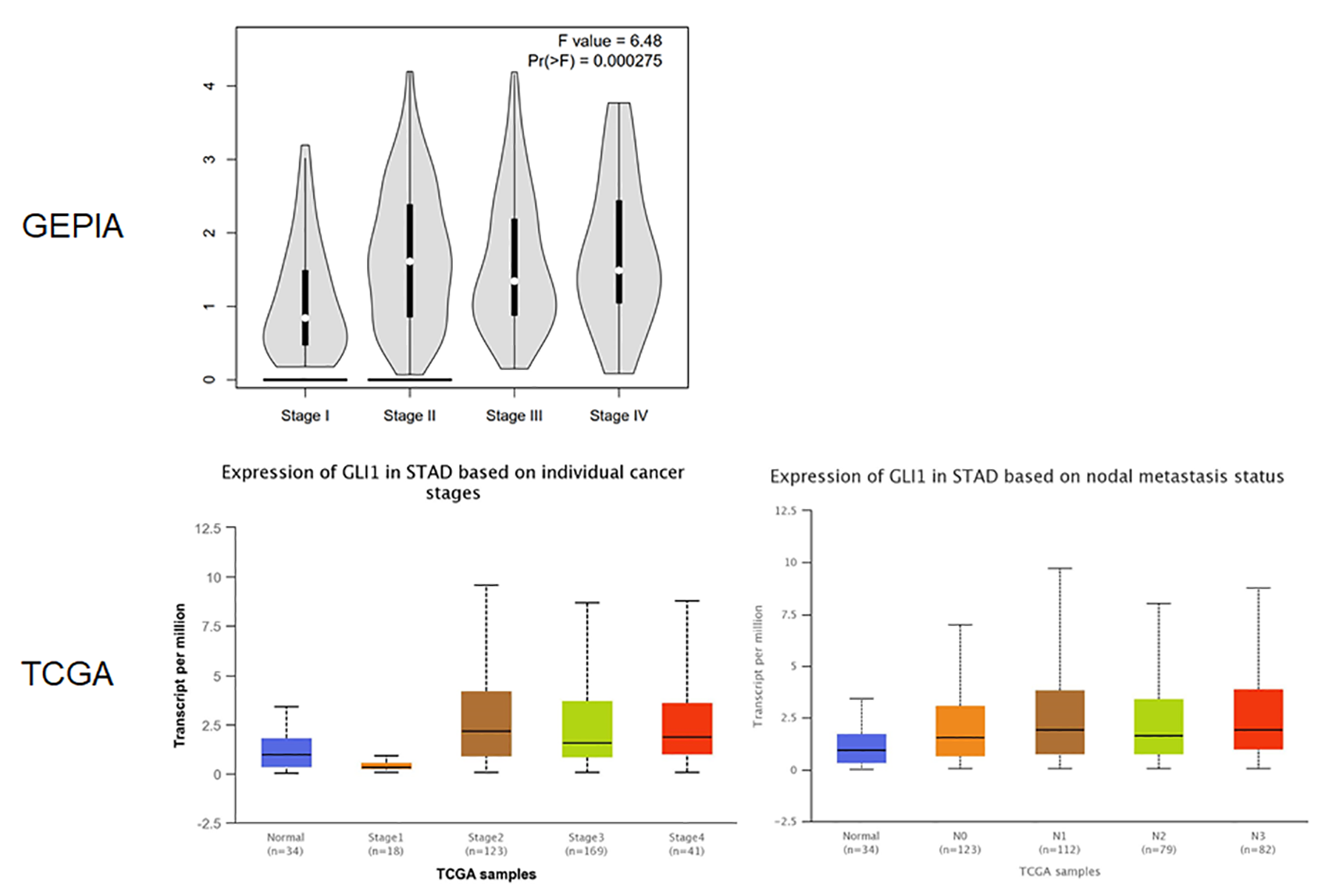

Supplement: Supplementary file 1 — Additional file 1: Supplemental Figure. The association between Gli1 mRNA expression and clinical stage, lymph node metastasis in GEPIA and TCGA data in GA. [file 13000_2020_949_MOESM1_ESM.tif]
